# From brief encounters to lifelong unions

**DOI:** 10.7554/eLife.01893

**Published:** 2013-12-24

**Authors:** Bradley JSC Olson

**Affiliations:** 1 **Bradley JSC Olson** is in the Division of Biology, Kansas State University, Manhattan, United Statesbjsco@k-state.edu

**Keywords:** opisthokonts, *Capsaspora*, alternative splicing, cell differentiation, evolutionary transitions, multicellularity, Other

## Abstract

Could the transient aggregation of unicellular organisms have paved the way for the evolution of the multicellular animals?

**Related research article** Sebé-Pedrós A, Irimia M, del Campo J, Parra-Acero H, Russ C, Nusbaum C, Blencowe BJ, Ruiz-Trillo I. 2013. Regulated aggregative multicellularity in a close unicellular relative of metazoa. *eLife* **2**:e01287. doi: 10.7554/eLife.01287

**Image** During the life cycle of the unicellular amoeboid *Capsaspora owczarzaki*, individual cells gather to form a multicellular aggregate

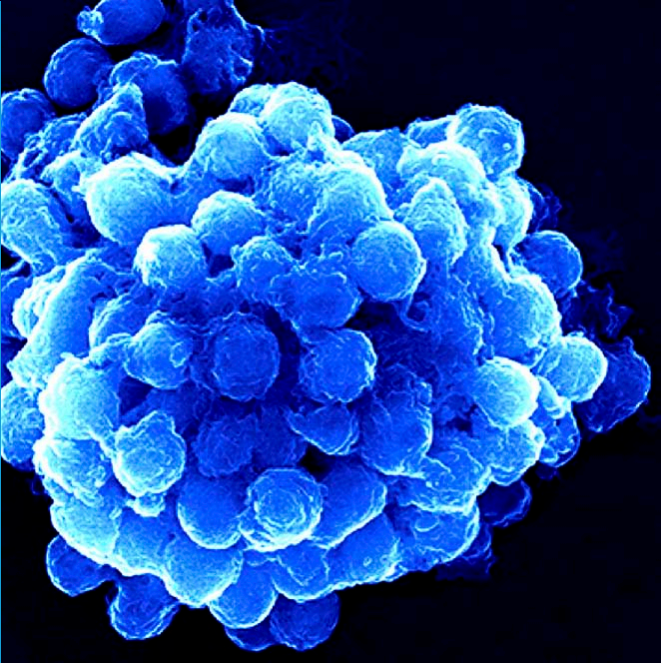

When multicellular organisms developed from unicellular ancestors, it was a major evolutionary transition (Maynard Smith and Szathmary, 1995). Multicellular life is thought to have evolved by two mechanisms—clonal development or aggregative development (Grosberg and Strathmann, 2007; Figure 1)—but we are just beginning to understand its genetic basis.

**Figure 1.**
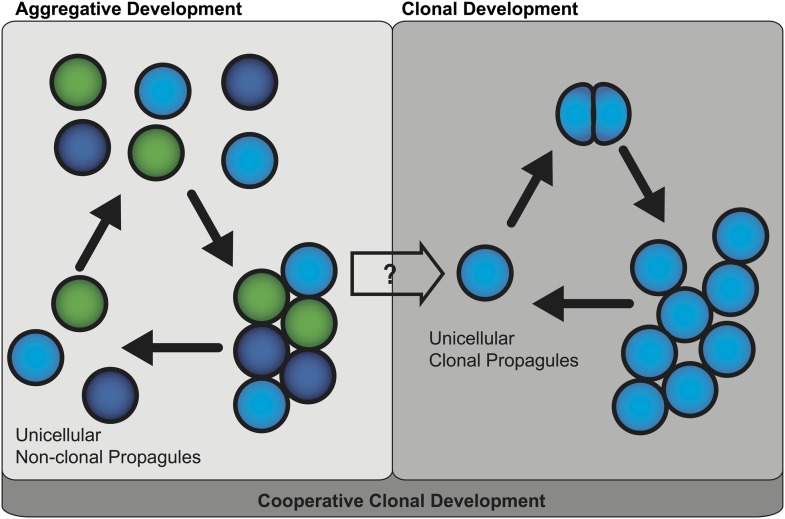
Two mechanisms of multicellular evolution. On the left, organisms that evolved multicellularity by aggregative development have a life cycle where individuals from the environment aggregate, and cooperate to form a multicellular organism. These cells need not be genetically identical (indicated by the different colors). In many organisms with this life cycle, only some cells are dispersed for reproduction. On the right, organisms that evolved multicellularity by clonal development remain attached together after each cell division, forming groups of undifferentiated cells. Each cell in the group can produce a genetically identical reproductive cell, or ‘propagule’, that produces genetically uniform offspring. If aggregative development and clonal development are both important for multicellular evolution in metazoa, as the results of Sebé-Pedrós et al. suggest, then a new unified mechanism—‘cooperative clonal development’—is required.

In plants and animals, multicellularity is thought to have evolved as a result of clonal development (King, 2004; Rokas, 2008). Here, ancient unicellular organisms evolved methods of cell-cell adhesion that prevented their cells from fully separating after cell division. At first these organisms resembled clumps of undifferentiated cells, but later cells within these organisms specialized, allowing the evolution of complex and differentiated tissues. Significantly, the organisms that evolved multicellularity by clonal development must go through a single cell stage every generation, which means that all the cells in each multicellular organism are genetically identical.

Aggregative development, on the other hand, is thought to be the less common mechanism because it is typically observed in organisms with unusual life cycles, such as the slime molds and slime bacteria (Bonner, 2000; Rokas, 2008). Aggregative development involves previously free-living, single cells gathering together and cooperating to form a multicellular organism. As such, the cells in the resultant organism may not necessarily be genetically identical. Moreover, in most organisms that undergo aggregative development, only subsets of cells are dispersed for reproduction. This means that these organisms do not always have to go through the ‘unicellular bottleneck’ that limits genetic diversity during clonal development (Grosberg and Strathmann, 2007).

One potential disadvantage of aggregative development is that individual cells can act selfishly or ‘cheat’ to ensure they are selected for reproduction, even if this reduces the fitness of the multicellular organisms as a whole. Because of this, it is assumed that the cost of actively preventing cheating in aggregative organisms limits their potential to evolve complex tissues and organs (Bonner, 2000). As such, the unicellular bottleneck is considered important for the evolution of complex tissues in plants and animals (Grosberg and Strathmann, 2007). Now Iñaki Ruiz-Trillo of the Institut de Biologia Evolutiva and the University of Barcelona, Benjamin Blencowe of the University Toronto and co-workers—including Arnau Sebé-Pedrós as first author—have challenged this assumption by examining the life cycle of a close unicellular relative of the multicellular animals or ‘metazoa’, *Capsaspora owczarzaki* (Sebé-Pedrós et al., 2013).

Metazoans evolved from a primitive amoeba- or fungal-like unicellular organism between about 0.8 and 1 billion years ago. Although very few relatives of these unicellular pre-metazoans exist today (King, 2004; Rokas, 2008), the choanoflagellates were amongst the first to be recognized as such (King, 2004). Representative organisms are found as either unicellular organisms, or in clonal groups called ‘rosettes’. Since these multicellular rosettes are formed by cell division in which the daughter cells do not separate, this has been considered to support a clonal development origin for metazoan. However, the recent discovery that choanoflagellate multicellularity is influenced by compounds produced by a symbiotic bacteria suggests that our understanding of the events that lead to animal multicellularity may be incomplete (Alegado et al., 2012).

Recently, a new group of amoeba-like organisms that are slightly more distant relatives of the metazoa were discovered (Steenkamp et al., 2006; Ruiz-Trillo et al., 2007, 2008; Shalchian-Tabrizi et al., 2008; Torruella et al., 2012; Suga et al., 2013). The genome of one of these species, *Capsaspora owczarzarki*, was sequenced and was found to contain several families of proteins that were thought to absent in unicellular pre-metazoans (King et al., 2008). This means that these protein families were most likely present in the ancestors of the metazoans, but have subsequently been lost in the choanoflagellates. Indeed with the completion of genome sequences for organisms occupying the lower branches of the metazoan family tree, a short list of gene families required for the evolution of multicellular animals is now available (Suga et al., 2013). However, these comparative genomics studies have yet to determine the specific genes that were required for the first steps toward clonal multicellularity.

Now Sebé-Pedrós, Ruiz-Trillo, Blencowe and colleagues—who are based in Barcelona, Toronto and the Broad Institute—report the surprising discovery that this species has an aggregative life cycle, not a clonal life cycle as expected. Sebé-Pedrós et al. have also identified a small subset of genes that are linked to aggregative multicellularity. *Capsaspora* multiplies as amoeba-like cells with small finger-like projections, called filipodia, and these allow the cells to move across a surface in search of nutrients. After the filopodial stage, and presumably when nutrients are exhausted, the cells follow one of two developmental fates. In some cases cells retract their filopodia, detach from the surface and form individual cysts. However, cells can follow an alternative pathway where they randomly aggregate, and produce a thickened extracellular matrix that holds them together. These multicellular aggregates can later break down into individual cysts, possibly aiding in dispersal. The discovery that a close unicellular relative of metazoa has an aggregative life cycle stage challenges the idea that clonal development led to the evolution of multicellular metazoans.

Sebé-Pedrós et al. also compared the gene expression profiles of these three life cycle stages. Filopodial cells show the expected signs of cell proliferation; cysts, on the other hand, show a signature of cell starvation and signs of entering into a dormant state. However, the aggregative cells express genes that are required for cell-cell adhesion and cell-cell communication, but are not expressed during the other life cycle stages. Furthermore, as the specific proteins involved in cell-cell adhesion and cell-cell communication may interact with each other, this suggests that these two pathways might have co-evolved. If future work shows that these two pathways did co-evolve, it suggests a strong link between them and the evolution of aggregative development.

Thus these findings question whether clonal development and aggregative development are really two distinct mechanisms of multicellular evolution. One possible scenario for the evolution of multicellular metazoa is a combination of these two mechanisms, or ‘cooperative clonal development’ (Figure 1). In this scenario when, environmental conditions became adverse, unicellular pre-metazoans were able to cooperate and form aggregates of similar cells. At some point, cooperative aggregation became a selective advantage that led to more permanent cell-cell adhesion. Subsequently, clonal development became a selective advantage by short-circuiting reproductive dispersal of individual cells. Once clonal development evolved, genetic uniformity was selected for through a single cell bottleneck. The evolution of clonal development thus provided a selective advantage in controlling cheating, thus allowing the evolution of complex developmental patterns that are the hallmark of animal diversity present today.
